# “DerMohscopy”: utility of dermoscopy combined with Mohs micrographic surgery for the treatment of basal cell carcinoma^☆^

**DOI:** 10.1016/j.abd.2020.11.012

**Published:** 2022-01-07

**Authors:** Felipe Bochnia Cerci, Stanislav N. Tolkachjov, Betina Werner

**Affiliations:** a Post-graduate Program in Internal Medicine and Health Sciences, Universidade Federal do Paraná, Curitiba, PR, Brazil; b Clínica Cepelle, Curitiba, PR, Brazil; c Epiphany Dermatology, Dallas, TX, United States; d Department of Pathology, Hospital de Clínicas da Universidade Federal do Paraná, Curitiba, PR, Brazil

Dear Editor,

Many Mohs surgeons perform dermoscopy as an auxiliary method for demarcating surgical margins, especially for poorly defined tumors clinically. In such cases, dermoscopic findings are often subtle and theoretically impossible to remember if not documented. Dermoscopic mapping has been recently described to allow correlation of dermoscopic and histopathological findings, especially in cases with positive histological margins.^1^

Due to the practicality of capturing high-quality images with portable equipment, we incorporated dermoscopic mapping of the surgical margins as part of Mohs micrographic surgery (MMS), creating DerMohscopy. Although dermoscopy has been used as an adjunct method for initial MMS margin evaluation for more than ten years, results are divergent regarding its ability to decrease the number of stages.^2^, ^3^, ^4^, ^5^, ^6^ Previously published studies focused on the comparison of naked eye versus dermoscopic margin delineation for tumor removal. The key focus of the current study, however, is to illustrate how the combination of MMS and dermoscopy may be used as a learning tool for the Mohs surgeon through dermoscopic mapping and documentation.

The selected cases exemplify potential utilities of DerMohscopy, such as the correlation of dermoscopic and histopathological findings when margins are positive, the ability to better delineate a second stage based on preoperative dermoscopic findings, and improvement of classic and non-classic dermoscopic criteria knowledge of distinct BCC subtypes through mapping of the debulking specimen.

Traditionally, in MMS, the delineation of the tumor and the orientation markings are performed with the same colored pen. To allow an accurate correlation between dermoscopy and histopathology, two additional steps are required:

1 – Make the orientation markings on the patient with colored pens of the same color used to ink the surgical specimen (Fig. 1). This allows faster and easier correlation of dermoscopy with histopathology, especially for tumors that do not “fit” in one dermoscopic photograph.

**Figure 1:**
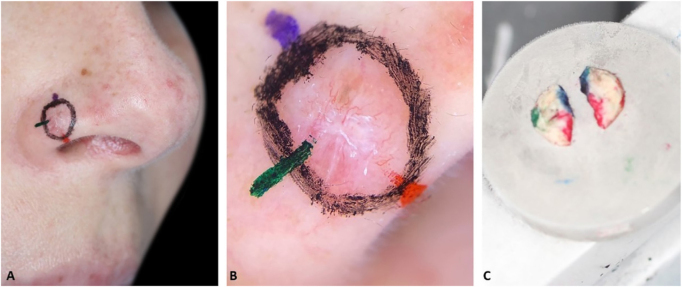
DerMohscopy. (A), Colored orientation marks. (B), Photographic documentation of the mapped dermoscopy. (C), Surgical specimen is inked with the same colors as the markings on the patient.

2 – Document dermoscopy with a cell phone camera (Fig. 1B).

After these steps, MMS is performed with the standard technique.

Fig. 2 demonstrates the anticipation of possible subsequent stages based on preoperative dermoscopy. Dermoscopic documentation and its correlation with histopathology allowed the Mohs surgeon to confidently resect a second stage larger than usual (7 mm instead of the usual 1–2 mm), avoiding multiple subsequent stages. In the perioperative period, a new demarcation based on dermoscopy would be impossible because the mechanical compression of the local anesthetic and the vasoconstrictor effect of epinephrine would mask some dermoscopic BCC criteria. The second stage included the entire dotted area, nonetheless, the third stage with a 2 mm margin was required for complete tumor removal. This conservative approach on the first stage can be valid for ill-defined tumors located in cosmetically sensitive areas where telangiectasias and sebaceous glands are common such as in the present case.

**Figure 2:**
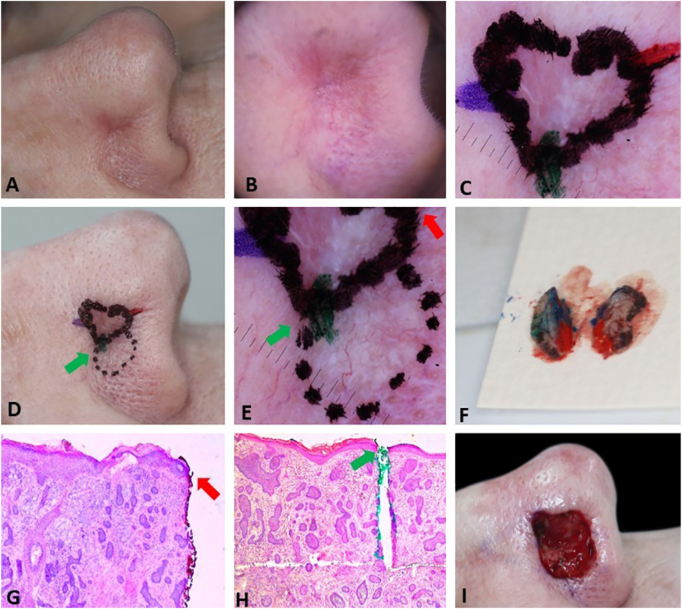
Preoperative demarcation of suspicious areas based on dermoscopy. (A–B), Ill-defined infiltrative BCC. (C), First stage margins. (D–E), Suspicious area marked with a dotted line in case it was necessary to remove it. F, Surgical specimen inked. (G–H), BCC on the green and red markings (Hematoxylin & eosin, ×25). (I), Final surgical defect.

Fig. 3 exemplifies the correlation of dermoscopic and histopathological findings when margins are positive. Dermoscopic mapping allowed a “re-analysis” of dermoscopy and correlation with histopathology in this ill-defined BCC on a rhinophymatous nose. White, red structureless areas are a “non-classic” criterion for BCC but are present in about 40% of cases.^7^ In tumors affecting the nose with numerous sebaceous glands and many telangiectasias, where the distinction between normal skin and classic BCC dermoscopic findings (arboriform telangiectasias, for example) is even more challenging, the non-classic findings such as red, white structureless areas may be helpful.

**Figure 3:**
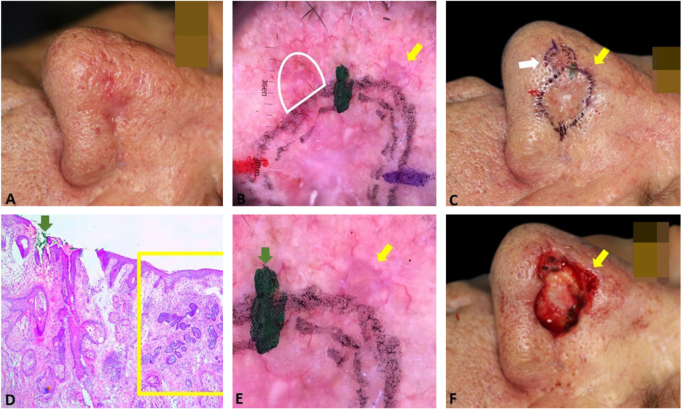
Correlation of dermoscopic and histopathological findings when margins are positive. (A), Ill-defined BCC. (B), Dermoscopic mapping. After a careful assessment, the white area was included in the first stage margin. (C), First stage margins. (D), Positive lateral margin (yellow rectangle) adjacent to the green marking (Hematoxylin & eosin, ×25). (E), The yellow arrow indicates the area corresponding to the positive histopathologic margin; with white, red structureless areas on dermoscopy. (F), Final defect. The yellow arrow indicates the area removed in the second stage.

Fig. 4 illustrates the debulking mapping. In addition to the dermoscopic mapping of the margins, debulking was mapped to correlate tumor dermoscopic criteria with the histopathologic subtype. The mapping of the debulking also allows the Mohs surgeon to evaluate if the tumor demarcation was not “beyond the necessary”. Debulking processing can also be performed with horizontal sections.

**Figure 4:**
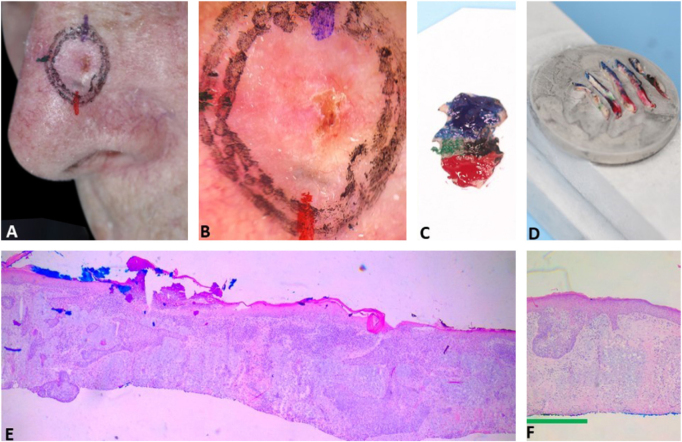
Debulking mapping. (A), Ill-defined BCC. (B), Dermoscopy of the tumor. (C), Debulking mapped similarly to dermoscopy. (D), Debulking vertical sections prior to inclusion. (E–F), Histopathology showed predominantly infiltrative BCC in the blue area and superficial subtype in the green area (Hematoxylin & eosin, ×40).

Mohs surgeons deal with ill-defined facial BCCs on a daily basis, often located in anatomic locations with many sebaceous glands and numerous telangiectasias and photodamage. These characteristics make the distinction between tumor and healthy skin more challenging than in other parts of the body.^8^

Dermoscopy, like any diagnostic exam, has a learning curve. DerMohscopy allows an almost immediate correlation of dermoscopy and histopathologic findings provided by MMS, being a learning opportunity for Mohs surgeons in these challenging cases. The complete analysis of surgical margins allows confirmation of dermoscopy-based markings, in addition to providing other learning opportunities in identifying second stage margins, histopathologic subtypes, classic and non-classic dermoscopic features, as exemplified. We know that dermoscopy does not replace the complete histopathological analysis of the surgical margins but it is traditionally a diagnostic tool.^9^ It constitutes an auxiliary method for the demarcation of clinically ill-defined BCCs, which often have non-classical dermoscopic criteria on the periphery, difficult to observe on clinical inspection alone.^10^

It is important to emphasize that when demarcating a tumor, not only the dermoscopic findings should be considered, but also clinical inspection, palpation, and skin traction. Furthermore, during dermoscopy, not only the presence of telangiectasias but also their pattern and associated dermoscopic findings are essential for delineating the tumor from healthy tissue.^7^

The disadvantage of DerMohscopy is the additional time required, which with practice, can be done in a few minutes. If one is not in a busy practice, it is worth investing time for a continuous dermoscopic improvement of the Mohs procedure, which may be useful when dealing with aggressive and/or ill-defined facial BCCs.

## Financial support

None declared.

## Authors’ contributions

Felipe Bochnia Cerci: Participation in the design and planning of the study; collection, analysis, and interpretation of data; writing; critical review of the manuscript; approval of the final version.

Stanislav Tolkachjov: Writing; critical review of the manuscript; approval of the final version.

Betina Werner: Writing; critical review of the manuscript; approval of the final version.

## Conflicts of interest

None declared.
